# Dose-response relationship between different physical activity types and depressive symptoms in middle-aged and elderly people: a study of Chinese urban-rural differences

**DOI:** 10.1186/s12889-025-23128-x

**Published:** 2025-05-26

**Authors:** Rui Wang, Jianyu Tan, Zhewei Su, Yiting Kong, Su Hong, Pan Ran, Yuancen Zhong, Li Kuang

**Affiliations:** 1 Psychiatric Center, The First Affiliated Hospital of Chongqing Medical University, Yuzhong District, Chongqing, 400016 China; 2 Department of Psychiatry, The First Affiliated Hospital of Chongqing Medical University, Chongqing, China

**Keywords:** Depressive symptoms, Physical activity, Dose, Urban-rural difference

## Abstract

**Background:**

Evidence on the dose-response connection involving depressive symptoms and various types of physical activity in urban and rural populations is scarce. This study compares the dose-response connection across depressive symptoms and different types of activity in populations living in rural and urban areas.

**Methods:**

The study is based on a cross-sectional weighted sample from the 2020 China Health and Retirement Longitudinal Study (CHARLS). Physical activity and depressive symptoms were assessed using self-reported activity from the CHARLS questionnaire and the Centre for Epidemiological Studies Depression Scale. Physical activity was computed in metabolic equivalents (METs) and depressive symptom cutoff is 10. 1 MET is the amount of oxygen used during rest (3.5 mL O2/kg/min). Multivariate logistic regression analyses were used to determine the dose-response relationship between different types of physical activity and depressive symptoms.

**Results:**

Physical activity of different types showed a varying relationship with depressive symptoms with dose in rural populations. However, in urban populations, recreational physical activity exhibited a negative correlation with depressive symptoms, while non-recreational activity displayed a positive correlation. Specifically, in the rural population, 600-3,000 METs-min/week of recreational physical activity was inversely linked to depressive symptoms, but recreational physical activity exceeding 3,000 METs-min/week was associated with a 13.2% risk increase in depressive symptoms (OR = 1.132, 95% CI: 1.131–1.132), and over 6,000 METs-min/week of non-recreational activity was similarly associated with an increased risk of depressive symptoms. In the urban population, recreational physical activity was related to a reduced incidence of depressive symptoms, with a maximum reduction of 71.7% at doses up to 9,000–12,000 METs min/week (OR = 0.283, 95% CI:0.282–0.284), and 6,000–9,000 non-recreational physical activities, the odds of increased risk of depressive symptoms were highest (OR = 2.224, 95% CI:2.221–2.227).

**Conclusions:**

Our study found a non-linear dose correlation between physical activities and depressive symptoms. The dose-response connection between different physical activity types and depressive symptoms varies between rural and urban areas. Recreational physical activity is recommended for urban and reasonable amounts of nonrecreational physical activity for rural populations to reduce the risk of depressive symptoms. However, causal interpretations cannot be made due to the cross-sectional design of the study.

**Supplementary Information:**

The online version contains supplementary material available at 10.1186/s12889-025-23128-x.

## Introduction

The 2019 Global Burden of Diseases report states that depression has played a major part in the disease burden of mental diseases. By 2030, WHO believes it’s going to be the highest-burden disease globally [1, 2].

Among middle-aged and elderly people, depressive symptoms are the main cause of emotional suffering, leading to an increase in physical illnesses, a heightened risk of daily activities and cognitive impairment, and even the rate of suicide, thereby significantly reducing their quality of life [3–5]. In China, the symptoms of depression differ significantly between urban and rural areas, with middle-aged and older people living in rural areas having more instances of depression than those in urban areas [6, 7]. Therefore, it is crucial to find targeted strategies tailored to the urban-rural disparities to effectively prevent middle-aged and older persons from developing depression.

Physical activity (PA) might prevent depression from starting by neurobiological (e.g., neuroplasticity, inflammation, or oxidative stress) and psychosocial mechanisms (e.g., self-esteem, self-concept, and stress tolerance) [8–10]. PA can be divided into recreational and non-recreational depending on the reason for doing it. Recreational physical activity (RPA) refers to physical activity undertaken for relaxation or exercise, while non-recreational physical activity (NRPA) generally refers to work-related physical activity [11, 12]. However, the correlation between the type and dose of PA and depressive symptoms remains controversial. A meta-analysis that included 15 studies revealed that individuals can derive significant mental health effects from physical activity when the dose of PA reaches the minimum recommended target dose, however, the potential benefit diminishes as the dose of activity increases [13]. WHO guidelines make a point that 600 METs-min/week is the suggested lowest dose of PA [14], but there is disagreement about the maximum level of PA that would be beneficial to individuals [15, 16]. Fernandez-Montero et al. did find an association between reduced risk for depression and greater levels of RPA [17], while other studies had argued that it was moderate-intensity RPA that was associated with psychological well-being, rather than high-intensity RPA [18, 19]. More heterogeneous association than recreational physical activity between NRPA and depression. Studies have indicated a beneficial correlation between mental health disorders and work-related PA [20], but the research on adults in Europe showed that work-related physical activity correlated to a decreased incidence of depression [21]. However, no association was found between NRPA and depression among older adults [11]. These inconsistencies highlight the need for further research to clarify the dose-response relationships between different types of PA and depressive symptoms.

Urban-rural differences in PA also exist. A study of 28 European Union countries found that over a 15-year period, inactivity rates grew in urban as well as rural regions relative to the baseline period, but the rise was larger in rural regions [22]. Furthermore, compared to urban areas, rural areas have lower incidence of RPA and higher incidence of NRPA [23, 24]. Despite these differences, there are limited studies that have explored how the type and dose of PA influence depressive symptoms, throughout rural and urban contexts, particularly among middle-aged and older adults. Studies found as age increased, participation in PA significantly decreased [25–27], however, it remains obscure which types and doses of PA are most beneficial to reduce symptoms of depression among middle-aged and older persons [28].

Urban-rural disparities in PA patterns and depressive symptoms are particularly pronounced among middle-aged and older adults. Understanding how different types and doses of PA affect depressive symptoms in urban and rural contexts is crucial for developing targeted interventions that address the specific needs of these populations. Despite the growing body of research on PA and depression, several gaps remain. First, most studies have treated PA as a homogeneous construct, failing to differentiate between RPA and NRPA, which may have distinct effects on mental health. Second, few studies have explored the dose-response relationship between PA and depressive symptoms in urban and rural populations, particularly among middle-aged and older adults. Our study aims to fill these gaps by examining the dose-response relationship between different types of PA and depressive symptoms while accounting for urban-rural disparities.

Based on existing evidence, we hypothesized that different types of PA show a different dose-response relationship with depressive symptoms and that this relationship would differ between urban and rural populations. To test these hypotheses, the study aimed to: (1) examine the dose-response connection between various types of PA versus depressive symptoms; and (2) investigate urban-rural variations in this relationship.

## Methods

### Study population

The China Health and Retirement Longitudinal Study (CHARLS), a follow-up survey of individuals 45 years of age and older in mainland China, provided data for this study. The CHARLS project used the multi-stage probabilities proportional to size sampling design. The sample covered 150 districts and 450 villages, involving more than 17,000 people at baseline. Its baseline survey was undertaken in 2011, with four rounds of regular questionnaires conducted in 2013, 2015, 2018, and 2020. All respondents used a face-to-face computer-assisted personal interview. The complete explanation of CHARLS has already been discussed previously [29]. This study used the latest data of CHARLS in 2020. CHARLS was approved by the Ethical Review Board of Peking University (IRB 00001052–11015) and all participants provided informed consent.

**Fig. 1:**
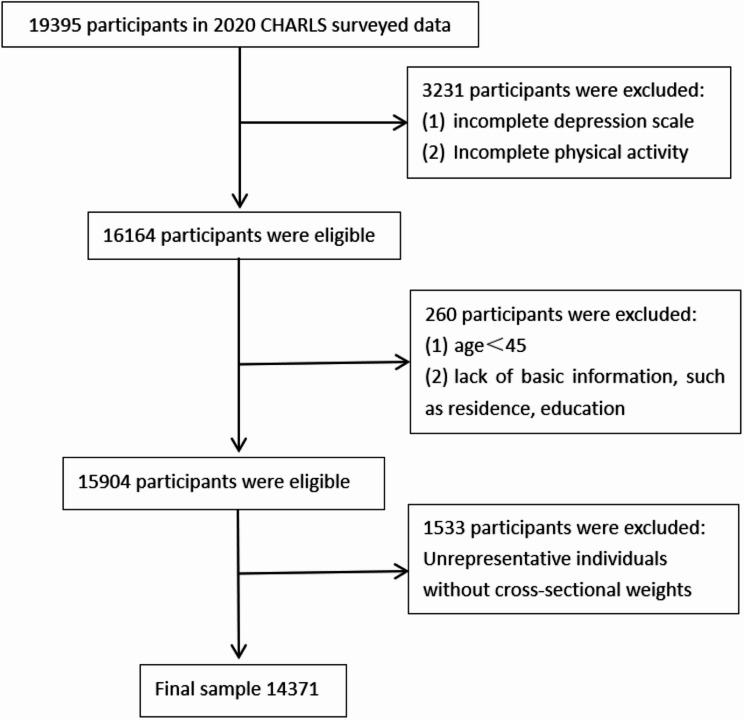
Study participants exclusion flowchart

The study sample comprised 19,395 individuals in 2020. We excluded non-representative individuals lacking weights, those who did not report physical activity levels and depressive symptoms, those who were younger than 45 years old, and those who lacked basic information such as place of residence, and education level. The higher percentage of excluded people are female, rural residents, and have no depressive symptoms (supplementary Table 1 for comparison of included and excluded participants). Ultimately, the analytic weighted sample was 528,467,093 (unweighted sample = 14,371) with complete information (Fig. 1).

### Measurement

#### Depressive symptoms

The 10-item Center for Epidemiologic Studies Depression Scale (CESD-10) was used to measure depressive symptoms, which was designed to screen for depressive symptoms in the general population and has good predictive accuracy compared to the full-length 20-item version of the CES-D [30]. In our study, the scale showed good internal consistency in both urban and rural populations (urban: Cronbach α = 0.750; rural: Cronbach α = 0.784) populations. The scale comprises 10 symptom items, each with four options for the past week: (1) Rarely or None (< 1 day); (2) Some or A Little (1–2 days); (3) Occasionally or Moderate Amount of Time (3–4 days); (4) Most of the Time (5–7 days). Prior studies have validated the CESD-10 in middle-aged and elderly adults in China, we scored the responses following previous studies [31]. The overall score spans from 0 to 30, with higher scores representing more severe depressive symptoms. Individuals scoring below 10 on the CESD-10 were deemed to exhibit no depressive symptoms, whereas a score of 10 or above indicated the presence of depressive symptoms in the individual [30].

#### Physical activity

The measurement of physical activity (PA) was conducted using five inquiries from the CHARLS 2020 questionnaire. The questionnaire used was similar in structure and description to the International Physical Activity Questionnaire (IPAQ), a globally recognized tool for collecting data on physical activity [32]. Previous studies have explained the difference between the two scales [33]. In this questionnaire, physical activity was categorized into three levels: vigorous physical activity (VPA) (e.g., activity that makes you breathe fast, such as aerobic exercise, fast cycling, etc.), moderate physical activity (MPA) (e.g., activity that makes you breathe a little faster than usual, such as lifting something light, mopping the floor, etc.), and low-intensity physical activity (LPA) (e.g., walking). The duration of each PA was classed into 5 levels, including no activity, 10–30 min/d, 30–119 min/d, 120–239 min/d and ≥ 240 min/d. The frequency of activity was 0–7 days per week, reasons for doing physical activity included job demands, entertainment, exercise, and more.

The participants underwent a comprehensive inquiry regarding the specifics of their physical activities, including the intensity, duration, frequency, and motivations. To standardize the analysis, we substituted the duration of each physical activity’s intensity with its median value. Since the physical activity time of more than 4 h can’t be taken as the median value, it was replaced with 4 h in this study [33]. To apply our findings more effectively, we employed metabolic equivalent (MET) to calculate physical activity intensity. Previous studies have shown that 1 MET is the amount of oxygen used during rest (3.5 mL O2/kg/min), while VPA, MPA, and LPA can each be expressed as 8 MET, 4 MET, and 3.3 MET [34]. The dose of PA = duration*frequency*MET (METs-min/week), the total physical activity (TPA) is determined by adding the scores for VPA, MPA, and LPA in METs-min/week [34]. Metabolic equivalent (MET) minutes per week were calculated for recreational and non-recreational physical activity in the research based on the reason for performing the physical activity. Recreational physical activities (RPA) are those carried out for entertainment or exercise, while non-recreational physical activities (NRPA) are those carried out for job demands or other purposes [35]. In this study, the dose of TPA = RPA + NRPA. Based on WHO guidelines [14], the minimal dose of PA needed for wellness is 600 METs-min/week, and referred to the classification of existing studies [36, 37], in this study we categorized the METs-min/week of physical activity into 7 categories based on multiples of the recommended dose (0-599,600–1199,1200–2999,3000–5999,6000–8999,9000–11999,≥12000).

#### Covariates

Based on the results of previous studies, we selected factors affecting depressive symptoms and physical activity as covariates to increase the stability of the regression model. The covariates included age, gender, education (< high school,≥high school), marital status (married, separate or divorced, widowed, never married), residence (rural or urban), alcohol consumption (more than once a month, less than once a month, none), smoking (still smoke, quit, never smoked), and chronic disease (no, yes).

### Statistical analyses

We used Individual Weight with household and Individual response adjustment to adjust for the unbalanced distribution of the urban and rural samples and used the weighted samples for statistical analyses. Individual weights are provided by CHARLS and this weight is used to correct for nonresponse and sampling-frame errors [29]. In the demographic data, Counts (percentages) were used to represent categorical variables, and means (standard deviation) were used to represent continuous variables. Using chi-square tests for categorical data and t-tests of variance for continuous variables, between-group differences in populations living in rural and urban areas were studied. Multivariate logistic regression was used to determine the odds ratios for depressive symptoms in relation to the 7 categories of physical activity. There were three models in the study: model 1 was unadjusted; model 2 was adjusted for gender and age; model 3 was adjusted for factors in model 2 plus alcohol consumption, smoking habits, marital status, education level, chronic illnesses, and residence. To look into potential differences in the association between PA and depressive symptoms across residences, we further divided the data by residence.

#### Sensitivity analyses

To assess the robustness and reliability of our findings, two sensitivity analyses were conducted. First, we performed an analysis using unweighted samples to examine the potential impact of weighting on the results. Second, missing data were handled through multiple imputation techniques, followed by the application of weights, to evaluate the effect of different strategies for managing missing data on the results.

All statistical analyses were performed with SPSS 25.0 and the results were visualized with R program (version 4.4.2).

## Results

### Sample features

The participants’ sociodemographic data is displayed in Table 1. The majority of study participants were from rural areas (65.81% of the weighted sample). The participants were 61.49 years old on the mean (SD = 9.51), with almost 50% of them being female. Among the participants, 85.6% were married and 82.5% had attained education level below high school level. 60.8% had never consumed alcohol and 60.0% had never smoked, while 36.2% suffered from chronic illnesses. Depressive symptoms were found in 35.7% of all participants, 26.8% of those in urban regions, and 40.3% of those in rural areas.

**Table 1:** Sociodemographic characteristics according to depressive symptoms in a weighted sample

Characteristics	Total	Urban	Rural	*P*-value
	(*n* = 528,467,093)	(*n* = 180,665,011)	(*n* = 347,802,082)	
**Age (years**,** M ± SD)**	61.49 ± 9.51	61.76 ± 9.76	61.36 ± 9.37	<0.001
**Gender (%)**				<0.001
Male	49.2	48.5	49.5	
Female	50.8	51.5	50.5	
**Marital status (%)**				<0.001
Married	85.6	85.7	85.6	
Separated or divorced	1.7	2.6	1.2	
Widowed	12.1	11.4	12.5	
Never Married	0.5	0.3	0.6	
**Education (%)**				<0.001
< High school	82.5	65.3	91.4	
≥High school	17.5	34.7	8.6	
**Alcohol consumption (%)**				<0.001
Drink More than Once a Month	28.7	30.8	27.5	
Drink But Less than Once a Month	10.5	13.2	9.1	
None of These	60.8	56.0	63.3	
**Smoking (%)**				<0.001
Still smoke	26.0	22.0	28.1	
Quit	14.0	14.7	13.7	
Never Smoked	60.0	63.3	58.3	
**Chronic disease (%)**				<0.001
No	63.8	62.6	64.4	
Yes	36.2	37.4	35.6	
**Depressive symptoms (%)**				<0.001
No	64.3	73.2	59.7	
Yes	35.7	26.8	40.3	

M ± SD: Mean ± standard deviation

Chronic disease includes: Hypertension, Dyslipidemia, Diabetes or High Blood Sugar, Cancer or Malignant Tumor, Chronic Lung Diseases, Liver Disease, Heart Problems, Stroke, Kidney Disease, Stomach or Other Digestive Disease, Emotional, Nervous, or Psychiatric Problems, Memory-Related Disease, Arthritis or Rheumatism, Asthma

### Disparities in physical activity in middle-aged and elderly people between rural-urban areas

According to Table 2, similar percentages of rural-urban populations participate in PA of more than 600 METs-min/week (urban:87.6%; rural:84.3%). However, there is a large difference in participation in different types of physical activity between rural-urban populations. Compared to the less than 600 METs-min/week group, The urban population has a higher rate of participation in the RPA (61.0%), while the rural population has a higher rate of participation in the NRPA (58.3%).

**Table 2:** Characteristics of type and dose of physical activity

Type and dose of Physical activity (METs-min/ week)	Total (*n* = 528,467,093)	Urban (*n* = 180,665,011)	Rural (*n* = 347,802,082)	*P*-value
**Total Physical activity (%)**				<0.001
0 to <600	14.6	12.4	15.7	
600 to <1200	4.9	4.9	4.9	
1200 to <3000	24.1	32.8	19.5	
3000 to <6000	22.4	28.4	19.2	
6000 to <9000	11.9	11.3	12.2	
9000 to <12,000	7.6	5.8	8.6	
≥ 12,000	14.6	4.5	19.9	
**Recreational Physical activity (%)**				<0.001
0 to <600	51.4	39.0	57.9	
600 to <1200	5.4	6.0	5.1	
1200 to <3000	24.4	31.8	20.5	
3000 to <6000	12.7	16.8	10.5	
6000 to <9000	3.2	4.1	2.7	
9000 to <12,000	1.7	1.9	1.7	
≥ 12,000	1.3	0.6	1.7	
**Non-recreational Physical activity (%)**				<0.001
0 to <600	47.1	57.5	41.7	
600 to <1200	3.3	3.6	3.1	
1200 to <3000	12.8	15.9	11.2	
3000 to <6000	12.4	12.0	12.6	
6000 to <9000	7.1	5.4	8.0	
9000 to <12,000	5.2	2.0	6.8	
≥ 12,000	12.0	3.5	16.5	

Percentages may not add up to 100% due to rounding

### Correlation across various doses of physical activity with depressive symptoms in the total population

**Fig. 2:**
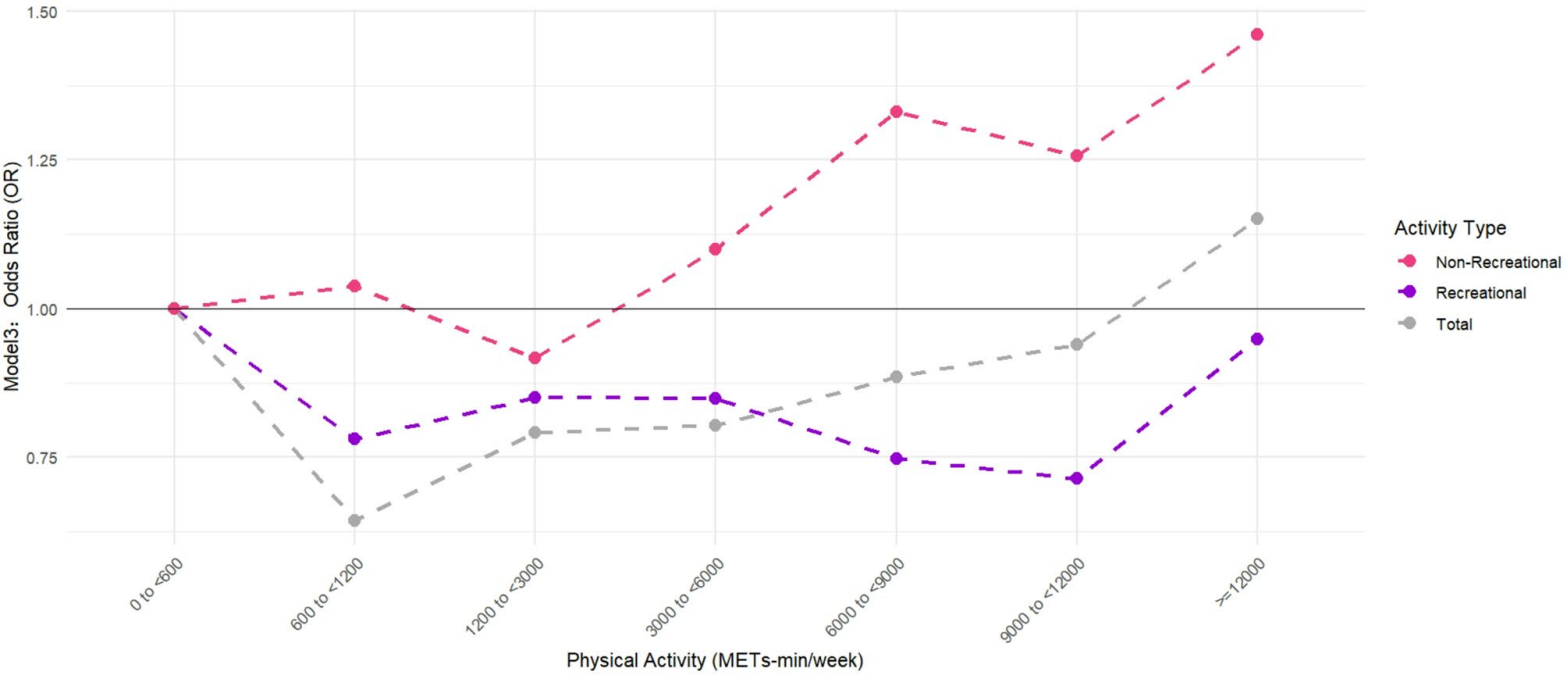
OR for physical activity and depressive symptoms in the total population

Table 3 presents the multivariate logistic regression analysis results, model 3 is the most important model because it includes the fullest adjustment for covariates. In all models, In comparison to the group regarded as inactive (0 to<600 METs-min/week), engaging in TPA at the lowest suggested dose of PA (600 METs-min/week), was related to the notably decreased incidence of depressive symptoms. And when the TPA dose was increased to more than 12,000 METs-min/week, the potential for depressive symptoms increased as well (Table 3). This relationship changed when TPA was categorized into recreational and non-recreational based on the reason for PA (Fig. 2). RPA remained related to a reduced risk of depressive symptoms, which at doses up to 9,000–12,000 METs min/week reached a peak reduction of 28.5% (Table 3: model 3). However, when engaging in NRPA, the benefit of PA in preventing the onset of depressive symptoms almost entirely disappeared. And when the NRPA dose exceeded 10 times the recommended dose, it was substantially connected to an elevated risk of depressive symptoms. At NRPA doses beyond the 20 times suggested (≥ 12000 METs min/week), the positive correlation with depressive symptoms reached a peak of 46.1% (Table 3: model 3).

**Table 3:** Relationships between physical activity and depressive symptoms in a weighted sample

Physical activity (METs-min/ week)	model 1				model 2				model 3		
	OR	95% CI	*P*-value		OR	95% CI	*P*-value		OR	95% CI	*P*-value
total Physical activity											
0 to <600	1				1				1		
600 to <1200	0.632	0.631–0.632	<0.001		0.617	0.616–0.617	<0.001		0.643	0.642–0.644	<0.001
1200 to <3000	0.681	0.680–0.681	<0.001		0.689	0.689–0.690	<0.001		0.792	0.792–0.793	<0.001
3000 to <6000	0.687	0.686–0.687	<0.001		0.704	0.704–0.705	<0.001		0.803	0.802–0.803	<0.001
6000 to <9000	0.817	0.817–0.818	<0.001		0.843	0.842–0.843	<0.001		0.885	0.885–0.886	<0.001
9000 to <12,000	0.859	0.859–0.860	<0.001		0.927	0.927–0.928	<0.001		0.939	0.938–0.940	<0.001
≥ 12,000	1.060	1.059–1.061	<0.001		1.229	1.229–1.230	<0.001		1.151	1.150–1.152	<0.001
recreational Physical activity											
0 to <600	1				1				1		
600 to <1200	0.768	0.768–0.769	<0.001		0.737	0.736–0.737	<0.001		0.781	0.780–0.782	<0.001
1200 to <3000	0.797	0.796–0.797	<0.001		0.759	0.758–0.759	<0.001		0.850	0.850–0.850	<0.001
3000 to <6000	0.795	0.795–0.796	<0.001		0.742	0.742–0.743	<0.001		0.849	0.849–0.850	<0.001
6000 to <9000	0.694	0.693–0.694	<0.001		0.671	0.670–0.672	<0.001		0.747	0.746–0.748	<0.001
9000 to <12,000	0.715	0.714–0.716	<0.001		0.658	0.657–0.659	<0.001		0.715	0.714–0.716	<0.001
≥ 12,000	1.043	1.041–1.045	<0.001		0.976	0.974–0.977	<0.001		0.948	0.946–0.949	<0.001
non-recreational Physical activity											
0 to <600	1				1				1		
600 to <1200	1.037	1.036–1.038	<0.001		1.042	1.041–1.043	<0.001		1.038	1.037–1.039	<0.001
1200 to <3000	0.876	0.875–0.876	<0.001		0.896	0.896–0.897	<0.001		0.916	0.916–0.917	<0.001
3000 to <6000	1.055	1.054–1.055	<0.001		1.139	1.138–1.140	<0.001		1.100	1.099–1.101	<0.001
6000 to <9000	1.318	1.317–1.318	<0.001		1.445	1.444–1.446	<0.001		1.331	1.330–1.332	<0.001
9000 to <12,000	1.301	1.300-1.302	<0.001		1.484	1.483–1.485	<0.001		1.257	1.256–1.258	<0.001
≥ 12,000	1.434	1.433–1.435	<0.001		1.738	1.737–1.739	<0.001		1.461	1.460–1.462	<0.001

CI = Confidence Interval; OR = Odds Ratio. METs: metabolic equivalents

Model 1, without adjustment; Model 2, adjusted for gender and age; Model 3, adjusted for variables in model 2 plus marital status, smoking, drinking, education, chronic disease and residence

### Disparities in the connection between depressive symptoms and different physical activity doses in urban and rural middle-aged and elderly people

**Fig. 3:**
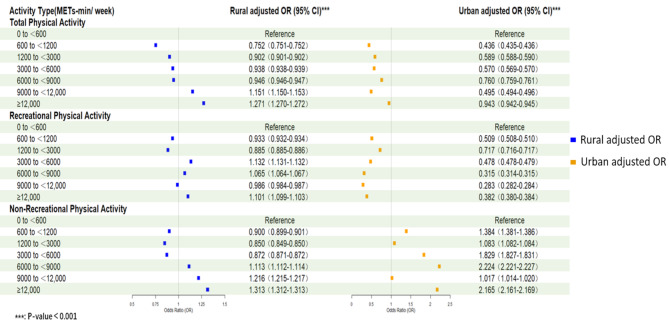
Adjusted relationships between physical activity and depressive symptoms, stratified by Residence

Given the distinctions in symptoms of depression comparing urban and rural populations, to examine the role that dwelling plays in modulating the connection between the dose of PA and depressed symptoms, we stratified the data by residency. Figure 3 shows the association between depressive symptoms and TPA, RPA, and NRPA among those living in urban and rural populations. TPA and RPA doses were negatively associated with depressive symptoms in urban populations. Specifically, with a maximum reduction of 56.5% at TPA doses of 600-1,200 METs min/week and 71.7% at RPA doses of 9,000–12,000 METs min/week. Whereas in rural populations, this association varied with dose. TPA doses of 600-9,000 METs-min/week and RPA doses of 600-3,000 METs-min/week were protective against depressive symptoms. However, TPA and RPA doses above 9,000 METs-min/week and 3,000 METs-min/week increased the risk of depressive symptoms by 15.1% and 13.2%, respectively (Fig. 3). For NRPA, the risk of depressive symptoms was increased in urban populations at doses up to 600 METs-min/week (OR = 1.384, 95% CI: 1.381–1.386), whereas in rural populations, the increased risk did not occur until doses reached 6,000–9,000 METs-min/week (OR = 1.113. 95% CI: 1.112–1.114), and this is when the risk of depressive symptoms is maximal in the urban population, with a 122.4% increase in risk. The risk of depressive symptoms peaked at NRPA doses above 12,000 METs-min/week, with a 31.3% increase in the rural population (Fig. 3).

To verify the robustness of the findings, we compared the results of the weighted sample principal analysis (Fig. 3), the unweighted sample analysis (Supplementary Table 2), and the weighted sample analysis after multiple imputations (Supplementary Table 3). In summary, the trends in the results from the three analyses were generally consistent, showing a nonlinear dose-response relationship between depressive symptoms and physical activity. Confidence intervals for unweighted analyses were commonly wider than those for weighted analyses, probably because weighted analyses reduce sampling error and thus improve the precision of estimates. The results of the weighted analyses after multiple imputations differ slightly from the results of the main analyses in the magnitude of the ORs, which may be related to the characteristics of the missing data that were imputed when interpolation. Nonetheless, the trend of the results from both weighted and unweighted analyses was consistent, further supporting the robustness of the findings.

## Discussion

This study demonstrated that individuals in rural and urban areas did different types and doses of physical activity. Participants in urban areas are more prone to RPA, while those in rural areas prefer NRPA. Firstly, Economic factors play an important role, with wealthier people more likely to engage in RPA and inadequate NRPA than those with lower incomes [38, 39]. Urban middle-aged and older adults have more time for RPA because they are under less economic pressure and have greater financial security. However, those in rural areas have lower disposable income, and they may increase their income by increasing their working hours and strength, which will lead to less time for RPA and excessive NRPA [26, 40]. Secondly, Because of technology advancements, urbanization may result in a rise of sedentary jobs thus a reduction for NRPA among the urban population, Manual labor in agriculture is an important source of livelihood in rural areas [41, 42]. Thirdly, urban areas offer more facilities such as gyms, parks, and community activity centers, increasing opportunities for recreational activities. Rural areas, however, lack such facilities and organized opportunities may lead to a greater inclination among rural residents to pursue productive work-related and home-based activities, rather than RPA projects [43, 44]. Furthermore, urban communities offer a diverse range of social activities and a broader social network, providing opportunities for elderly individuals to engage with like-minded individuals in shared activities. Rural areas are characterized by a shortage of support systems, a reduction in social activities, with an increased dependence on family [45].

Our study found that PA reduced the likelihood of depressive symptoms, but the risk for it increases when the dose of physical activity exceeds 12,000 METs-min/week. In a study of 99,846 Korean adults, those who performed 1–15 times (600 to < 9,000 METs-min/week) the recommended dose of PA had a statistical reduction in the chance of depression versus the sedentary population, while those who performed more than 12,000 METs-min/week PA had an increased [34], and our results agreed with Kim et al., suggesting a potential universal threshold for the mental health benefits of PA. Excessive physical activity can also contribute to becoming depressed, even if it is for recreational purposes [18]. This similarity may be attributed to shared biological mechanisms underlying the relationship between PA and depression. For instance, PA can improve depression by improving neuroplasticity, inflammation, and oxidative stress levels in depressed patients [46]. Specifically, physical activity increases BDNF levels [47] and promotes neuroplasticity, which enhances emotion regulation. Additionally, moderate PA reduces systemic inflammation by lowering levels of pro-inflammatory cytokines, such as IL-6 and TNF, which are often elevated in individuals with depression [48–50]. However, vigorous PA can negatively affect the immune system, leading to a reduction in the body’s antioxidant defenses and an increase in adrenocorticotropic hormone levels, which can adversely affect depression [51, 52]. These common mechanisms may explain why both studies observed a non-linear relationship between PA dose and depressive symptoms. Therefore, we suggested excessive physical activity should be avoided to prevent depressive symptoms.

Our study additionally suggested that there were rural-urban disparities in the non-linear dose-response correlation of depressive symptoms and PA, as well as such differences across types of physical activity. In urban populations, TPA and RPA had antidepressant protective effects that varied in magnitude depending on the activity dose, with maximum protective effects at doses of 600-1,200 for TPA and 9,000–12,000 for RPA. Antidepressant symptomatic doses of TPA and RPA ranged from 600 to 9,000 METs min/week and 600-3,000 METs min/week in rural populations, respectively, and this protective effect is absent at doses above the range, even translating into increased risk. The risk spectrum for RNPA was over 600 METs min/week in urban and over 6,000 METs min/week in rural populations. Overall, moderate RPA may decrease the risk of depressive symptoms, but NRPA has the opposite effect, consistent with the earlier findings [53, 54]. This may be due to the fact that NRPA is associated with higher psychological stress responses, whereas RPA tends to be associated with lower psychological stress [55] Also NRPA was linked with a significantly higher risk of insomnia, and insomnia symptoms tended to be bi-directionally related to depressive symptoms [56, 57]. According to certain research, the positive impacts of PA on depressive symptoms have been found only among urban populations, with no beneficial influences affecting depressive symptoms for rural individuals [37, 58, 59]. For this study, we found that among middle-aged and older rural persons, PA did not completely eliminate the benefits for depressive symptoms, although this range was very narrow. This discrepancy may be due to several factors. First, we more carefully divided the PA doses in our study, whereas in other studies with urban-rural differences, PA was only roughly divided into high, medium, and low intensity, which may mask some of the benefits. By using a more granular approach to PA dose categorization, we identified a narrower but still significant range of PA benefiting rural populations. Second, the preceding study [37] combined physical types without considering the distinct motivations may have led to an underestimation of the mental health benefits of PA in rural populations. This distinction is crucial because RPA and NRPA may differ significantly in motivation, psychological experiences, and effects on mental health. For example, our study found a stronger protective antidepressant effect from moderate NRPA than from moderate RPA in rural areas, a result that further supports the need to differentiate between RPA and NRPA. We speculate that psychological factors may be responsible for the variations in NRPA and RPA dose-response and depressive symptoms between rural and urban areas. PA may produce some psychosocial advantages that could lessen the symptoms of depression. Previous studies have indicated that low self-efficacy might be linked to higher levels of depressive symptoms and that PA can increase self-efficacy, but work-related PA may be perceived as a mandatory task, and therefore feelings of joy and self-efficacy may not be present [20, 60]. Furthermore, life stress is a major risk factor for depressive symptoms and RPA can disperse stress, but NRPA does not, and work itself can be a source of stress [20, 61]. Long-term chronic stress impairs adaptive structural plasticity within interconnected brain regions leading to depressive symptoms [62]. However, in rural areas, middle-aged and elderly people have the stronger inclination to actively increase their working hours to increase their income, so they may not see NRPA as a mandatory task and a source of stress [40]. As a result, people in rural areas may be more adaptable to and benefit more from NRPA than those in urban areas, potentially narrowing the scope of NRPA risk.

Our study has several limitations. First, it should be noted that the exposure and outcome factors in this study were measured by self-report scales, thus recollection, social desirability, and information bias may have been present. Future studies could benefit from using objective measures, such as accelerometers, to enhance data accuracy. Second, since this study was cross-sectional, we were unable to completely understand the causal and bidirectional relationship between PA and depressive symptoms, we suggest that future studies could further validate the relationship using longitudinal designs or experimental studies. Third, although our study included a number of covariates, there were other factors that may have influenced the results that we did not include, such as the economic situation, psychosocial factors, access to mental health services, and environmental factors (e.g., availability of recreational facilities). These factors may potentially confound the relationship between physical activity and depressive symptoms by influencing both the exposure and the outcome, so they should be included in future studies to improve the internal validity of the findings and the understanding of the phenomenon. Fourth, In this study, we conducted subgroup analyses to compare the relationship between depressive symptoms and physical activity among urban and rural populations. In the statistical analyses, we did not take into account the effect of multiple comparisons, which may increase Error I; in addition, although we adjusted using individual sample weights to reduce selection bias and improve the representativeness of the results to the overall population, significant differences between included and excluded individuals on multiple characteristics may still affect the external validity of the study (Supplementary Table 1), so the results should be interpreted with caution. Finally, the classification of MET thresholds, while we referred to the existing studies, may not fully capture individual variability. Fixed thresholds, although widely used in population-level studies, might overlook nuances in how different individuals experience and report physical activity. Future studies could explore alternative categorizations of MET thresholds or incorporate more granular measures of physical activity to better capture individual variability. There are also some strengths in our study. It is the first study to look at both non-recreational and recreational activities in relation to the dose-response of depressive symptoms in a mainland Chinese population. This study also fills a gap in the field by describing fully the dose of PA in the elderly Chinese population and investigating what links PA and depressive symptoms in this population as well as urban-rural differences. Our findings suggest that the purpose as well as the dose of physical activity need to be taken into account when developing public health policies or interventions to reduce depressive symptoms through physical activity. Additionally, urban-rural differences in PA patterns should be considered to tailor interventions to the specific needs of each population.

## Conclusion

In summary, our study suggests that there is a non-linear dose correlation between distinct PA and depressive symptoms. Urban-rural populations had different ranges of distinct physical activity doses for antidepressant symptoms and for increased risk of depressive symptoms. The range and maximum threshold of antidepressant symptoms for recreational physical activity were greater in urban than in rural populations. Meanwhile, rural people have a narrower risk spectrum and stronger protective effect for NRPA participation than urban people. For middle-aged and elderly people, RPA is recommended for urban populations, and RPA of 1,200 to 3,000 METs min /week (e.g., 5–7 h of brisk walking or 3–4 h of jogging per week) or NRPA of 600-6,000 METs min /week (e.g., 2.5–20 h of agricultural work per week) for rural populations to prevent depressive symptoms. Excessive exercise (≥ 12,000 METs min/week, e.g., > 20 h of vigorous activity per week) increases the risk of depressive symptoms and should be avoided in middle-aged and older adults. These findings have important implications for public health interventions, suggesting the need for targeted physical activity guidelines for the urban and rural populations.

However, the cross-sectional design and reliance on self-reported PA data may affect the interpretation of results. Future studies should incorporate longitudinal designs, use objective measures of PA, and explore the role of economic and social factors in shaping PA patterns and mental health outcomes, particularly in rural populations. Additionally, investigating the mechanisms underlying the differential effects of RPA and NRPA on depressive symptoms could provide deeper insights for targeted interventions.

## Electronic supplementary material

Below is the link to the electronic supplementary material.

Supplementary Material 1

## Data Availability

The data for this study came from the China Health and Retirement Longitudinal Study, https://charls.pku.edu.cn.

## Abbreviations

CHARLS: China Health and Retirement Longitudinal Study
CI: Confidence interval
LPA: Low-intensity physical activity
MET: Metabolic equivalent
MPA: Moderate physical activity
NRPA: Non-recreational physical activity
OR: Odds ratio
PA: Physical activity
RPA: Recreational physical activity
SD: Standard Deviation
TPA: Total physical activity
VPA: Vigorous physical activity
WHO: World Health Organization
