# Supplementary material for: Dose-response relationship between different physical activity types and depressive symptoms in middle-aged and elderly people: a study of Chinese urban-rural differences
Source: BMC Public Health. 2025 May 26;25:1931. doi: 10.1186/s12889-025-23128-x (PMC12105178; doi:10.1186/s12889-025-23128-x)
Supplement: Supplementary file 1 — Supplementary Material 1 [file 12889_2025_23128_MOESM1_ESM.docx]

**Supplementary Table 1: Comparison of included and excluded participants.**

| **Characteristics** | **Included**  **(n=14371)** | **Excluded**  **(n=5024)** | **t/X²** | **P-value** |
| --- | --- | --- | --- | --- |
| **Age (years, M ± SD)** | 61.21±8.91 | 65.59±12.77 | 8.36 | ＜0.001 |
| **Gender (%)** |  |  | 40.35 | ＜0.001 |
| Male | 6931(48.2) | 2162(43.0) |  |  |
| Female | 7440(51.8) | 2862(57.0) |  |  |
| **Marital status (%)** |  |  | 200.29 | ＜0.001 |
| Married | 12356(86.0) | 3900(77.6) |  |  |
| Separated or divorced | 214(1.5) | 149(3.0) |  |  |
| Widowed | 1735(12.1) | 927(18.5) |  |  |
| Never Married | 66(0.5) | 48(1.0) |  |  |
| **Education (%)** |  |  | 0.11 | 0.736 |
| ＜High school | 12515(87.1) | 4338(86.9) |  |  |
| ≥High school | 1856(12.9) | 654(13.1) |  |  |
| **Alcohol consumption (%)** |  |  | 62.87 | ＜0.001 |
| Drink More than Once a Month | 3967(27.6) | 1126(22.6) |  |  |
| Drink But Less than Once a Month | 1408(9.8) | 427(8.6) |  |  |
| None of These | 8996(62.6) | 3425(68.8) |  |  |
| **Smoking (%)** |  |  | 46.97 | ＜0.001 |
| Still smoke | 3808(26.5) | 1103(22.3) |  |  |
| Quit | 1951(13.6) | 614(12.4) |  |  |
| Never Smoked | 8612(59.9) | 3233(65.3) |  |  |
| **Chronic disease (%)** |  |  | 46.75 | ＜0.001 |
| No | 9142(63.6) | 2923(58.2) |  |  |
| Yes | 5229(36.4) | 2101(41.8) |  |  |
| **Residence (%)** |  |  | 98.85 | ＜0.001 |
| rural | 11119(77.4) | 3528(70.4) |  |  |
| urban | 3252(22.6) | 1486(29.6) |  |  |
| **Depressive symptoms (%)** |  |  | 35.61 | ＜0.001 |
| No | 8853(61.6) | 1235(68.8) |  |  |
| Yes | 5518(38.4) | 559(31.2) |  |  |

**Supplementary Table 2: Adjusted relationships between physical activity and depressive symptoms, stratified by Residence in unweighted samples.**

| Physical activity (METs-min/ week) | rural | | |  | urban | | |  |
| --- | --- | --- | --- | --- | --- | --- | --- | --- |
|  | OR | 95% CI | P-value |  | OR | 95% CI | P-value |  |
| total Physical activity |  |  |  |  |  |  |  |  |
| 0 to ＜600 | 1 |  |  |  | 1 |  |  |  |
| 600 to ＜1200 | 0.823 | 0.666-1.017 | 0.072 |  | 0.709 | 0.471-1.067 | 0.099 |  |
| 1200 to ＜3000 | 0.907 | 0.793-1.038 | 0.156 |  | 0.603 | 0.468-0.777 | ＜0.001 |  |
| 3000 to ＜6000 | 0.894 | 0.782-1.022 | 0.102 |  | 0.571 | 0.438-0.744 | ＜0.001 |  |
| 6000 to ＜9000 | 1.003 | 0.864-1.165 | 0.965 |  | 0.696 | 0.512-0.944 | 0.020 |  |
| 9000 to ＜12,000 | 1.123 | 0.952-1.324 | 0.168 |  | 0.641 | 0.435-0.946 | 0.025 |  |
| ≥12,000 | 1.344 | 1.177-1.534 | ＜0.001 |  | 0.937 | 0.644-1.362 | 0.732 |  |
| recreational Physical activity |  |  |  |  |  |  |  |  |
| 0 to ＜600 | 1 |  |  |  | 1 |  |  |  |
| 600 to ＜1200 | 0.907 | 0.757-1.086 | 0.287 |  | 0.710 | 0.497-1.013 | 0.059 |  |
| 1200 to ＜3000 | 0.828 | 0.746-0.918 | ＜0.001 |  | 0.714 | 0.592-0.861 | ＜0.001 |  |
| 3000 to ＜6000 | 1.017 | 0.892-1.159 | 0.804 |  | 0.557 | 0.439-0.707 | ＜0.001 |  |
| 6000 to ＜9000 | 0.971 | 0.763-1.236 | 0.811 |  | 0.443 | 0.268-0.732 | 0.001 |  |
| 9000 to ＜12,000 | 1.041 | 0.766-1.414 | 0.799 |  | 0.352 | 0.179-0.692 | 0.002 |  |
| ≥12,000 | 1.416 | 1.029-1.949 | 0.033 |  | 0.496 | 0.177-1.392 | 0.183 |  |
| non-recreational Physical activity |  |  |  |  |  |  |  |  |
| 0 to ＜600 | 1 |  |  |  | 1 |  |  |  |
| 600 to ＜1200 | 0.986 | 0.778-1.248 | 0.904 |  | 1.388 | 0.941-2.048 | 0.099 |  |
| 1200 to ＜3000 | 0.946 | 0.827-1.083 | 0.422 |  | 1.060 | 0.835-1.346 | 0.631 |  |
| 3000 to ＜6000 | 0.960 | 0.846-1.090 | 0.530 |  | 1.596 | 1.241-2.052 | ＜0.001 |  |
| 6000 to ＜9000 | 1.244 | 1.071-1.445 | 0.004 |  | 1.586 | 1.147-2.194 | 0.005 |  |
| 9000 to ＜12,000 | 1.201 | 1.021-1.413 | 0.027 |  | 1.208 | 0.742-1.966 | 0.448 |  |
| ≥12,000 | 1.385 | 1.234-1.555 | ＜0.001 |  | 1.838 | 1.279-2.641 | 0.001 |  |

CI = Confidence Interval; OR = Odds Ratio. METs: metabolic equivalents.

adjusted for gender, age, marital status, smoking, drinking, education and chronic disease

**Supplementary Table 3: Adjusted relationships between physical activity and depressive symptoms, stratified by Residence in weighted samples after multiple interpolations.**

| Physical activity (METs-min/ week) | rural | | |  | urban | | |  |
| --- | --- | --- | --- | --- | --- | --- | --- | --- |
|  | OR | 95% CI | P-value |  | OR | 95% CI | P-value |  |
| total Physical activity |  |  |  |  |  |  |  |  |
| 0 to ＜600 | 1 |  |  |  | 1 |  |  |  |
| 600 to ＜1200 | 0.826 | 0.825-0.826 | ＜0.001 |  | 0.553 | 0.552-0.553 | ＜0.001 |  |
| 1200 to ＜3000 | 0.888 | 0.888-0.889 | ＜0.001 |  | 0.679 | 0.678-0.679 | ＜0.001 |  |
| 3000 to ＜6000 | 0.934 | 0.934-0.934 | ＜0.001 |  | 0.689 | 0.688-0.689 | ＜0.001 |  |
| 6000 to ＜9000 | 0.929 | 0.929-0.929 | ＜0.001 |  | 0.812 | 0.812-0.813 | ＜0.001 |  |
| 9000 to ＜12,000 | 1.160 | 1.159-1.160 | ＜0.001 |  | 0.606 | 0.606-0.607 | ＜0.001 |  |
| ≥12,000 | 1.270 | 1.270-1.270 | ＜0.001 |  | 1.067 | 1.067-1.068 | ＜0.001 |  |
| recreational Physical activity |  |  |  |  |  |  |  |  |
| 0 to ＜600 | 1 |  |  |  | 1 |  |  |  |
| 600 to ＜1200 | 0.946 | 0.946-0.946 | ＜0.001 |  | 0.598 | 0.597-0.598 | ＜0.001 |  |
| 1200 to ＜3000 | 0.882 | 0.882-0.882 | ＜0.001 |  | 0.773 | 0.773-0.774 | ＜0.001 |  |
| 3000 to ＜6000 | 1.097 | 1.097-1.097 | ＜0.001 |  | 0.568 | 0.567-0.568 | ＜0.001 |  |
| 6000 to ＜9000 | 1.127 | 1.127-1.128 | ＜0.001 |  | 0.409 | 0.409-0.410 | ＜0.001 |  |
| 9000 to ＜12,000 | 1.038 | 1.037-1.039 | ＜0.001 |  | 0.266 | 0.266-0.266 | ＜0.001 |  |
| ≥12,000 | 1.156 | 1.155-1.157 | ＜0.001 |  | 0.363 | 0.363-0.364 | ＜0.001 |  |
| non-recreational Physical activity |  |  |  |  |  |  |  |  |
| 0 to ＜600 | 1 |  |  |  | 1 |  |  |  |
| 600 to ＜1200 | 0.898 | 0.898-0.899 | ＜0.001 |  | 1.149 | 1.149-1.150 | ＜0.001 |  |
| 1200 to ＜3000 | 0.870 | 0.869-0.870 | ＜0.001 |  | 1.208 | 1.208-1.209 | ＜0.001 |  |
| 3000 to ＜6000 | 0.866 | 0.866-0.866 | ＜0.001 |  | 1.593 | 1.592-1.594 | ＜0.001 |  |
| 6000 to ＜9000 | 1.082 | 1.081-1.082 | ＜0.001 |  | 1.858 | 1.857-1.859 | ＜0.001 |  |
| 9000 to ＜12,000 | 1.205 | 1.205-1.206 | ＜0.001 |  | 1.181 | 1.180-1.182 | ＜0.001 |  |
| ≥12,000 | 1.300 | 1.300-1.301 | ＜0.001 |  | 2.047 | 2.045-2.048 | ＜0.001 |  |

CI = Confidence Interval; OR = Odds Ratio. METs: metabolic equivalents.

adjusted for gender, age, marital status, smoking, drinking, education and chronic disease
